# Neurocysticercosis on the Arabian Peninsula, 2003–2011

**DOI:** 10.3201/eid1901.120432

**Published:** 2013-01

**Authors:** Oscar H. Del Brutto

**Affiliations:** Author affiliation: Hospital-Clínica Kennedy, Universidad de Especialidades Espíritu Santo, Guayaquil, Ecuador

**Keywords:** cysticercosis, neurocysticercosis, Arabian Peninsula, Taenia solium, parasites

**To the Editor:** Neurocysticercosis occurs when humans become intermediate hosts of the tapeworm *Taenia solium* by ingesting its eggs after contact with a *Taenia* spp. carrier. This parasitic disease is endemic to most of the developing world, where it represents a leading cause of acquired epilepsy (*1*). In conjunction with an increasing number of immigrants from disease-endemic areas, there has been a recent increase in the number of patients with a diagnosis of neurocysticercosis in industrialized countries.

On the basis of the incorrect assumption that human neurocysticercosis does not occur in countries in which religious laws prohibit swine breeding and consumption of pork, the disease has been considered nonexistent in Muslim countries of the Arab world. However, sporadic cases were reported during the last 2 decades of the 20th century, mainly in immigrants from India, and several case series have suggested that the prevalence of neurocysticercosis in the Arab world has been increasing over the past few years.

A Medline and manual search of the literature identified 7 reports of 39 patients with neurocysticercosis on the Arabian Peninsula during 2003–2011 (*2*–*8*). Of these patients, 30 were from Kuwait, 5 from Saudi Arabia, and 4 from Qatar. Mean ± SD age of these patients was 16.9 ± 13.4 years (age range 2–44 years), and 25 (64%) were women. Twenty-four patients (62%) were <18 years of age. Seizures were the primary manifestation of neurocysticercosis in 35 (90%) patients. Two of the remaining patients had focal neurologic deficits, 1 had cognitive disease, and 1 had disease that was fortuitously discovered.

Neuroimaging studies showed parenchymal brain cysticercosis in the 39 patients that appeared as 1 or 2 enhancing lesions in 34 patients (colloidal cysts) and as vesicular cysts in 5 patients. Enzyme-linked immunoelectrotransfer blotting of serum detected antibodies against cysticerci in 12 (91%) of 23 patients tested. Twelve patients received cysticidal drug therapy and 7 biopsy specimens of brain lesions were obtained from 7 patients (Table). According to currently accepted diagnostic criteria, 32 patients had definitive neurocysticercosis and 7 had probable neurocysticercosis (*9*). Results of testing for *Taenia* spp. eggs in fecal samples from 3 patients were negative. In contrast, fecal examinations of household contacts of the 3 patients identified 1 carrier of *Taenia* spp.

**Table:** Characteristics of 39 patients with neurocysticercosis reported from countries on the Arabian Peninsula, 2003–2011*

Characteristic	Value
Mean age ± SD, y	16.9 ± 13.4
Sex, %	
M	36
F	64
Country	
Kuwait	30 (77)
Saudi Arabia	5 (13)
Qatar	4 (10)
Citzenship status	
Arabian Peninsula country	29 (74)
Immigrant from disease-endemic area	8 (21)
International traveler	1 (3)
Clinical manifestations	
Seizures	35 (90)
Focal neurologic deficit	2 (5)
Cognitive decrease	1 (3)
Asymptomatic	1 (3)
Form of disease	
1 or 2 parenchymal brain cysticercus granulomas	34 (87)
Vesicular parenchymal brain cysts	5 (13)

*Values are no. (%) except as indicated.

The 4 patients from Qatar were citizens of Qatar, and 4 of 5 patients from Saudi Arabia were citizens of this country; the other patient was an immigrant from India. One Qatari citizen had a history of traveling to disease-endemic areas, and 1 Saudi Arabian citizen had an immigrant housekeeper from a disease-endemic area. Of the 30 patients reported from Kuwait, 23 were Kuwaiti citizens and 7 were immigrants from disease-endemic areas (mainly India). Sixteen of the 23 Kuwaiti citizens had immigrants from disease-endemic countries working at their homes. Cases of neurocysticercosis in family members were reported by 7 persons.

This review suggests that the number of patients with neurocysticercosis on the Arabian Peninsula is increasing. Most cases were autochthonous, and many occurred in wealthy families who employed babysitters and housekeepers from disease-endemic areas. Although *Taenia* spp. eggs were not identified in most of these persons, it is likely that some were *Taenia* spp. carriers who infected persons for whom they worked through nonhygienic handling of food products or directly by the fecal–oral route.

Neurocysticercosis is a disease most often acquired from a human infected with *T. solium* tapeworms, and infected swine can perpetuate the infection (*1*). Although swine husbandry is not allowed on the Arabian Peninsula, *Taenia* spp. carriers who enter countries in this region every year might infect native persons and increase the prevalence of neurocysticercosis without infected swine.

The pattern of disease expression of neurocysticercosis in countries in this region is similar to that observed in patients from India, i.e., parenchymal brain cysticerci in the acute encephalitic phase (*10*). This benign form of the disease is characterized by development of 1 or 2 parenchymal brain cysts in the coloidal stage and occurs most often in persons who do not eat pork and who do not have contact with infected swine. Differential diagnosis with other infections of the nervous system is a problem with these patients. Proper interpretation of diagnostic criteria for neurocysticercosis will enable a correct diagnosis in most cases, obviating the practice of unnecessary surgical procedures (*9*).

The prevalence and incidence of neurocysticercosis on the Arabian Peninsula is unknown, and many cases might not have been reported. Compulsory reporting of cases will help determine the incidence and prevalence of parasitic disease. Also, identification of *Taeni*a spp. carriers among household contacts of neurocysticercosis patients will enable detection of potential sources of infection and reduce spread of this disease.
